# A serological screening for potential viral pathogens among semi-domesticated Eurasian tundra reindeer (*Rangifer tarandus tarandus*) in Finland

**DOI:** 10.1186/s13028-023-00671-4

**Published:** 2023-02-22

**Authors:** Morten Tryland, Cristina Wetzel Cunha, Boris Fuchs, Eva Marie Breines, Hong Li, Pikka Jokelainen, Sauli Laaksonen

**Affiliations:** 1 Department of Forestry and Wildlife Management, Inland Norway University of Applied Sciences, 2480 Koppang, Norway; 2 Department of Arctic and Marine Biology, UiT the Arctic University of Norway, Framstredet 39, Breivika, 9019 Tromsö, Norway; 3 Animal Disease Research Unit, US Department of Agriculture-Agricultural Research Service, Washington State University, Pullman, WA USA; 4 Infectious Disease Preparedness, Statens Serum Institut, Copenhagen, Denmark; 5 Faculty of Veterinary Medicine, University of Helsinki, Helsinki, Finland

**Keywords:** Alphaherpesvirus, Bluetongue virus, CvHV2, Gammaherpesvirus, Pestivirus, Schmallenbergvirus, Serology, Viral pathogens, Wildlife

## Abstract

**Background:**

Reindeer herding and husbandry is a traditional and important livelihood in Fennoscandia, and about 200,000 semi-domesticated reindeer are herded in Finland. Climatic changes, leading to ice-locked winter pastures, and encroachment of pasture-land have led to changes in reindeer husbandry, increasing the extent of supplementary or full ration feeding, which has become very common in Finland. Keeping reindeer in corrals or gathering them at permanent feeding sites will increase nose-to-nose contact between animals and they may be exposed to poor hygienic conditions. This may impact the epidemiology of infectious diseases, such as viral infections. The aim of this study was to investigate Finnish semi-domesticated reindeer for exposure to viral pathogens. Blood samples were collected from 596 reindeer (358 calves, 238 adults) in 2015, from nine reindeer slaughterhouses, representing most of the reindeer herding regions in Finland. Plasma samples were investigated for antibodies against a selection of known and potential reindeer viral pathogens by using enzyme linked immunosorbent assays (ELISA).

**Results:**

The screening suggested that alphaherpesvirus and gammaherpesvirus (malignant catarrhal fever virus group; MCFV) were enzootic in the reindeer population, with a seroprevalence of 46.5% (range at slaughterhouse level 28.6–64.3%) and 29.0% (range 3.5–62.2%), respectively. Whereas the seroprevalence was significantly higher for alphaherpesvirus among adult reindeer (91.2%) as compared to calves (16.8%), no age difference was revealed for antibodies against gammaherpesvirus. For alphaherpesvirus, the seroprevalence in the northernmost region, having the highest animal density (animals/km^2^), was significantly higher (55.6%) as compared to the southernmost region (36.2%), whereas the seroprevalence pattern for gammaherpesvirus indicated the opposite, with 8.1% in the north and 50.0% in the south. Four reindeer (0.7%) had antibodies against Pestivirus, whereas no antibodies were detected against Bluetongue virus or Schmallenbergvirus.

**Conclusions:**

Alphaherpesvirus and gammaherpesvirus (MCFV) seems to be enzootic in the Finnish reindeer population, similar to other reindeer herds in Fennoscandia, whereas the exposure to Pestivirus was low compared to findings in Norway and Sweden. The ongoing changes in the reindeer herding industry necessitate knowledge on reindeer health and diseases that may impact animal welfare and health of reindeer as well as the economy of the reindeer herding industry.

## Background

In Fennoscandia, reindeer herding and husbandry is a traditional and important livelihood which to a large extent is associated with the Sami people and culture. This is particularly the case in Sweden and Norway, with the exceptions of a few non-Sami owned herds in southern Norway. In Finland, however, reindeer herding is practiced by both Sami and non-Sami herders, since the eighteenth century when farmers in northern Finland learned reindeer herding from Sami herders [1]. Reindeer herding in Finland is conducted by 4394 registered reindeer herders in 54 reindeer herding cooperatives, which together comprise approximately 36% of the land area of Finland [2]. Supplementary feeding of reindeer, in corrals or by offering feed on pastures, has become common in Finland during winter months.

The reindeer population in Finland during 2004 to 2016 was on average 197,807 (range, 190,776–209,365) individuals, of which an average of 102,778 (range, 71,580–124,152) were slaughtered annually. Of the slaughtered reindeer, 77% were 6–8 months old calves. Approximately 74% of the Finnish reindeer were slaughtered in 19 EU-approved reindeer abattoirs, whereas the remaining animals were slaughtered in the field for private consumption and direct marketing [3–5]. In addition to important cultural dimensions and meat production, reindeer herding has additional economical values in terms of tourism, fur and leather production, use of bones and antlers for souvenirs, and the use of side products from slaughter in the pet food industry.

As for other wildlife and livestock species, reindeer are exposed to viral diseases. The reindeer alphaherpesvirus (Cervid herpesvirus 2; CvHV2, family *Herpesviridae*, genus *Varicellovirus*) has been found to be enzootic in most of the investigated reindeer and caribou herds in different countries [6, 7]. It has been shown that CvHV2 can be the transmissible pathogen for infectious keratoconjunctivitis (IKC) in reindeer [8] and that the virus also under experimental conditions was able to cause IKC [9]. Furthermore, CvHV2 is associated with infections of the respiratory tract, and the virus can be transferred vertically to the fetus and possibly contribute to abortion and reduced fitness of calves [10].

The disease malignant catarrhal fever (MCF) primarily affects wild and domestic ruminants and is caused by a group of gammaherpesviruses (MCFV-group). DNA specific for the gammaherpesviruses Ovine herpesvirus 2 (OvHV2) and Caprine herpesvirus 2 (CpHV2) has been detected in the central nervous system (CNS) of moose (*Alces alces*), roe deer (*Capreolus capreolus*) and red deer (*Cervus elaphus*) in Norway, associated with lesions indicating MCF [11]. OvHV2-specific DNA was also evident in the CNS of a semi-domesticated reindeer in Norway, displaying MCF-like clinical signs, such as hair loss and thickening of the skin with crusts on the muzzle, axillary regions and the distal parts of the legs. In addition, ocular signs like swollen eyelids, fibrinopurulent exudate and corneal opacity was evident [12]. In a previous screening for gammaherpesvirus antibodies among semi-domesticated reindeer (n = 3339) in Finnmark County, northern Norway, a seroprevalence of 3.5% was found [13].

*Pestivirus* is a genus in the *Flaviviridae* family, with four species causing disease in livestock; Pestivirus A (former Bovine viral diarrhea virus 1; BVDV-1), Pestivirus B (former BVDV-2), Pestivirus C (former Classical swine fever virus; CSFV), and Pestivirus D (former Border disease virus; BDV). In a screening of semi-domesticated reindeer (n = 618) in Norway (2013–2018), anti-Pestivirus antibodies were found in reindeer from five of eight investigated herds, with a mean seroprevalence of 19% in the seropositive herds [14]. In another serological screening of semi-domesticated reindeer from three herds in each country and about 40 reindeer from each herd (2016–2017), a seroprevalence of 48.5% and 41.2% was found in Sweden and Norway, respectively [15]. In contrast, a seroprevalence of 2.5% was found in Finland in the same study, but this study was based on relatively few animals from each country [15]. Antibodies against Pestivirus have also been detected in wild reindeer in Norway (4.2%) [16] and in different populations and subspecies of caribou (*R. t. granti*, *R. t. caribou* and *R. t. groenlandicus*) across Canada and in western Greenland [17].

Wild cervids are regarded as important in the epidemiology of Bluetongue virus (BTV; genus *Orbivirus*, family *Reoviridae*) [18, 19]. BTV also infects cattle and a wide range of other domestic animals. BTV has circulated in Europe since 2006, including southern parts of Sweden and Norway, but there are no indications of wild or semi-domesticated reindeer having been exposed to BTV.

Schmallenbergvirus (SBV; genus *Orthobunyavirus*, family *Bunyaviridae*) infects ruminants. The virus was first identified in Germany in 2011, spreading quickly to 27 European countries [20, 21], including Norway [22]. SBV is spread by biting midges (*Culicoides* spp.) and transplacental infection may result in abortion or the birth of malformed or weak offspring. Although a range of wildlife species is susceptible to SBV infection, including cervids [23], no evidence of exposure of reindeer in Fennoscandia has been reported.

The aim of this study, in which most of the reindeer herding regions of Finland were represented, was to investigate calves and adult semi-domesticated reindeer for exposure to selected viral pathogens.

## Methods

### Sampling

**Fig. 1:**
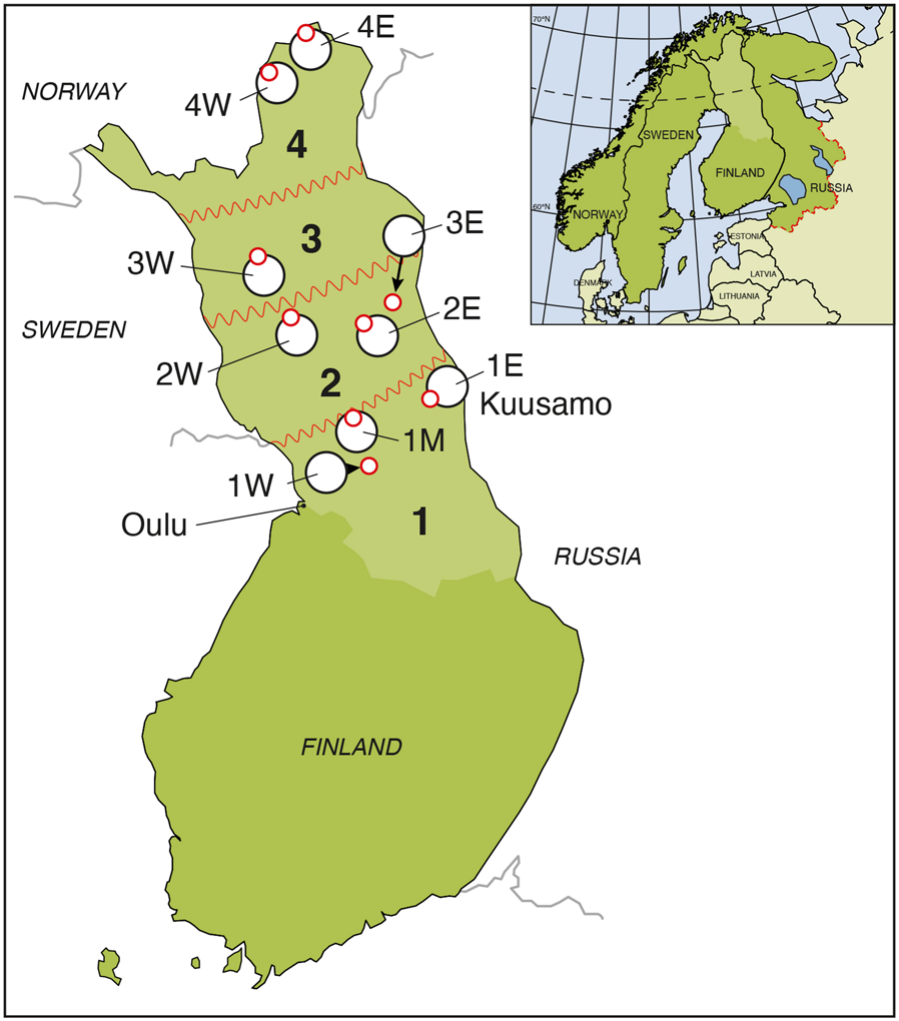
Map of Finland showing the location of the nine slaughterhouses where plasma samples were collected from altogether 596 semi-domesticated Eurasian tundra reindeer (*Rangifer tarandus tarandus*) in 2015. Within the four sampling regions (Region 1 to Region 4) from south to north, each slaughterhouse is representing a cooperative with several herds that are normally co-mingling. The slaughterhouses/cooperatives were designated according to the origin of the reindeer, from west to east (west, W; middle, M; east, E) within each Region. The light green area in the map represents the reindeer herding region in Finland (map adapted from [24])

Blood samples from 596 reindeer collected from September 30 to November 2, 2015, were analyzed in this study. Sex was not registered. Age was defined as calf (aged 6–7 months) or adult (> 18–19 months), and 358 calves and 238 adults were sampled. The samples were collected from nine different Finnish reindeer cooperatives and their slaughterhouses (Fig. 1). The cooperatives, containing several herds and owners, are thus representing the same geographical unit as the slaughterhouse. The overall geographical region was divided in four regions from south (Region 1) to north (Region 4) and the slaughterhouses were designated according to the origin of the reindeer [24], from west to east (west, W; middle, M; east, E) within each Region (Fig. 1). Blood samples were colleted directly into EDTA-tubes (Venosafe EDTA, Terumo, Belgium) during bleeding of the animals at the slaughterhouses, and transported chilled to the laboratory. Upon arrival, the samples were centrifuged (3000*g*, 15 min) and plasma were collected and stored frozen at − 20 °C until analysis.

### Serological analyses

All plasma samples were analyzed for antibodies against all the viruses investigated, using mainly commercial methods that all have been previously applied on blood samples from reindeer (Table 1).

**Table 1:** The serological tests used for detection of antibodies against selected viral pathogens in semi-domesticated Eurasian tundra reindeer (*Rangifer tarandus tarandus*) from nine slaughterhouses in Finland, 2015

Viral pathogen	Test and producer	Test principle	Antigen	References
Alphaherpesvirus	Laboratoire Service International, Lissieu, France	Blocking ELISA	Glycoprotein B	[25]
Gammaherpesvirus (MCFV-group)	Non-commercial	Competitive-inhibition ELISA	MCFV (AlHV-1) glycoprotein	[26, 27]
Pestivirus	SERELISA^®^ BVD p80 Ab Mono Blocking Synbiotics, Lyon, France	Blocking ELISA	p80	[28]
Bluetongue virus	ID Screen^®^ Bluetongue Competition ID Vet, Grabels, France	Competitive ELISA	Recombinant, VP7	[29]
Schmallenbergvirus	ID Screen^®^ Schmallenberg virus competition multispecies ID Vet, Grabels, France	Indirect ELISA	Recombinant SBV antigen	[30]

The samples were tested for anti-alphaherpesvirus antibodies using a commercial bovine enzyme-linked immunosorbent assay (ELISA) based on glycoprotein B (gB) from Bovine herpesvirus 1 (BoHV-1) from cattle as an antigen (Table 1). The ELISA has previously been validated against a virus neutralization test (VNT) for analyzing reindeer serum samples for anti-alphaherpesvirus antibodies [25]. The plasma samples were tested in 1:2 dilution (single well) and evaluated against bovine negative (NC) and positive (PC) control sera; bovine sera provided with the kit and samples obtained from reindeer experimentally inoculated with CvHV2 [31]. A competition percentage (C%) was calculated by dividing the optical density (OD) of the sample (OD S) by the mean OD of the bovine negative control (OD NC): C% = OD S/OD NC. Samples were categorized as positive if C% ≤ 0.5, doubtful if 0.5 < C% ≤ 0.55 and negative if C% > 0.55.

The samples were tested for the presence of specific antibodies to the MCFV-group of viruses by a direct competitive-inhibition ELISA (cELISA) [26, 27] (Table 1). The ELISA is based on a monoclonal antibody (15-A) directed against an epitope highly conserved among all MCFVs identified, including OvHV-2 and CpHV-2. The test was run as previously described for reindeer [13] (Table 1). Samples (50 μL) were tested in duplicates, using mean OD to evaluate the result. The sera were scored by mean OD. The performance of a plasma test sample was calculated as inhibition percentage (I%): I% = (100 − mean OD S × 100)/mean OD NC. A plasma sample was considered positive if I% ≥ 25 and negative if I% < 25. If substantially divergent results were generated between the duplicates, samples were rerun in duplicates. If divergent results between duplicates remained, the sample was classified as seronegative.

The plasma samples were tested for antibodies against Pestivirus using a commercial blocking ELISA designed for domestic ruminants (Table 1). The test is based on the p80/125 non-structural protein as antigen, which presumably is shared between all strains of Pestivirus A, B and D (former BVDV and BDV) [28]. This antigen has previously been used for testing reindeer for antibodies against Pestivirus and the ELISA kit has been validated against VNT [7, 15, 16, 32]. The plasma samples (S) were tested in a 1:10 dilution (single well). Positive (PC) and negative (NC) controls were provided with the kit. A competition percentage (%P) was calculated for each sample; (OD NC − OD S)/(OD NC − OD PC) × 100. A %P ≤ 30% was interpreted as being seronegative, 30 < %P < 50 as doubtful and ≥ 50% as being seropositive, in line with the manufacturer’s instructions for testing samples from bovines.

The plasma samples were tested for antibodies against BTV using a commercial competitive ELISA based on recombinant VP7 as antigen (Table 1). The antigen is conserved among BTV serotypes, and according to the producer is suitable for testing multiple species, including sheep, goat, buffalo, deer and others. The kit has previously been used for testing reindeer [33]. Samples (S) were tested undiluted (single well) and evaluated against positive (PC) and negative control (NC) sera provided with the kit. Optical density was read at 450 nm and results are expressed as competition percentage: S/N% = (OD S/OD NC) × 100. The results were classified according to the evaluation criteria and cut-offs provided: S/N% < 40 was considered positive and S/N% ≥ 40 was considered negative.

The plasma samples were investigated for antibodies against SBV using a commercial competitive ELISA based on recombinant SBV nucleoprotein antigens for multispecies testing (Table 1). The kit has previously been used for testing reindeer [33]. Plasma samples were tested undiluted (single well) and OD was read at 450 nm. Mean OD score was evaluated against the evaluation criteria and the PC and NC control sera provided by the manufacturer. The performance of the plasma samples was evaluated by calculating a competition percentage (S/N%): S/N% = (OD S/OD NC) × 100. Samples were classified as positive if S/N% ≤ 40, doubtful if 40% < S/N% ≤ 50%, and negative if S/N% > 50%.

Samples that were classified as doubtful (i.e., alphaherpesvirus, Pestivirus and BTV ELISAs) were retested.

### Statistical analyses

Logistic regression models were performed to estimate associations with testing positive for antibodies for each of the viruses with overall seroprevalence estimate of at least 10%, using R 1.4.1717 [34]. As potential explanatory variables we tested the geographical location (Region 1 to 4; Fig. 1, Table 3), the age group (calf/adult) and whether the sample tested positive for antibodies against another virus (i.e., indicating exposure to more than one of the viruses). Region and animal densities were confounded, with a south to north increase in animal density (no. of animals/km^2^), and we decided to use the region rather than animal density in the regression model. No observations with missing values in any predictor variable were included. We formulated a full model and removed non-significant variables one by one in a backward selection, starting with the highest P-value. Confidence intervals on the 95% level were calculated using the confint function depending on the R package MASS [35]. Model estimates and confidence intervals were calculated for each variable and then pointwise back transformed using the formula exp(^estimate^)/1 − exp(^estimate^).

## Results

Based on the OD read-outs from the ELISAs for the five different viruses and their interpretation criteria, the score for each test was calculated, which is depicted in Table 2.

**Table 2:** The score of each ELISA for the five different viruses is presented as range, mean and standard deviation (SD) based on the optical density read-outs and calculations for each test

ELISA	Range	Mean	SD
Alphaherpesvirus	0.01–2.20	0.46	0.35
Gammaherpesvirus (MCFV-group)	− 114.9 to 75.1	14.0	20.8
Pestivirus	− 68.8 to 97.6	− 2.3	21.8
Bluetonguevirus	11.5–158.9	113.5	16.0
Schmallenbergvirus	7.6–111.8	91.0	7.6

None of the samples tested positive for antibodies against BTV or SBV, and no samples were classified as doubtful for antibodies against BTV. The overall apparent seroprevalence estimates, i.e., the proportions of reindeer that tested positive for antibodies against alphaherpesvirus, gammaherpesvirus (MCFV-group) and Pestivirus, are summarized for each geographical region and at slaughterhouse level in Table 3. For Pestivirus and alphaherpesvirus, five and four plasma samples, respectively, were classified as doubtful. After a second test, they remained doubtful and were regarded as sero-negative in further calculations. For alphaherpesvirus and gammaherpesvirus, four and five samples, respectively, yielded divergent results between the duplicates also after being rerun, and these were classified as negative in further calculations. None of the animals investigated had detectable antibodies against BTV or SBV.

**Table 3:** Results from testing Finnish semi-domesticated Eurasian tundra reindeer for antibodies against alphaherpesvirus, gammaherpesvirus (MCFV-group) and Pestivirus, reporting number of seropositive animals (seroprevalence in percentage in parenthesis), with results for calves and adults at regional level (Region 1 = south to Region 4 = north)

Region	Animal density^1^: Reindeer/km^2^	Number of tested reindeer	Alphaherpesvirus	Gammaherpesvirus (MCFV-group)	Pestivirus
1W	0.76	56	16 (28.6%)	33 (58.9%)	0
1M	1.18	58	19 (32.8%)	15 (25.9%)	0
1E	1.68	74	33 (44.6%)	46 (62.2%)	0
Region 1 Total	1.21	188	C: 17/127 (13.4%) A: 51/61 (83.6%) Total: 68/188 = 36.2%	C: 60/127 (47%) A: 34/61 (56%) Total: 94/188 (50.0%)	C: 0/127 (0%) A: 0/61 (0%) Total: 0/188 (0%)
2W	1.77	63	25 (39.7%)	21 (33.3%)	1 (1.6%)
2E	1.25	66	32 (48.5%)	6 (9.1%)	0
Region 2 Total	1.51	129	C: 9/78 (11.5%) A: 48/51 (94.1%) Total: 57/129 (44.2%)	C: 19/78 (24.4%) A: 8/51 (15.7%) Total: 27/129 (20.9%)	C: 1/78 (0.01%) A: 0/51 (0%) Total: 1/129 (0.8%)
3W	1.39	57	20 (35.1%)	2 (3.6%)	1 (1.8%)
3E	2.28	98	63 (64.3%)	40 (40.8%)	1 (1.0%)
Region 3 Total	1.84	155	C: 17/83 (20.5%) A: 66/72 (92%) Total: 83/155 (53.5%)	C: 13/83 (15.7%) A: 29/72 (40.3%) Total: 42/155 (27.1%)	C: 2/83 (2.5%) A: 0/72 (0%) Total: 2/155 (1.3%)
4W	2.44	61	39 (63.9%)	5 (8.2%)	0
4E	2.37	63	30 (47.6%)	5 (7.9%)	1 (1.6%)
Region 4 Total	2.41	124	C: 17/70 (24.3%) A: 52/54 (96.3%) Total: 69/124 (55.6%)	C: 6/70 (8.6%) A: 4/54 (7.4%) Total: 10/124 (8.1%)	C: 0/70 (0%) A: 1/54 (1.9%) Total: 1/124 (0.8%)
Total	1.74	596	C: 60/358 (16.8%) A: 217/238 (91.2%) Total: 277/596 (46.5%)	C: 98/358 (27.4%) A: 75/238 (31.5%) Total: 173/596 (29.0%)	C: 3/358 (0.84%) A: 1/238 (0.42%) Total: 4/596 (0.67%)

^1^From [24]

Antibodies against alphaherpesvirus and MCFV-group of viruses were detected in samples from reindeer from all the slaughterhouses. The proportion of reindeer with antibodies against alphaherpesvirus was 46.5% (range at slaughterhouse level, 28.6–64.3%), and the proportion with antibodies against the MCFV-group was 29.0% (range at slaughterhouse level, 3.5–62.2%). Antibodies against Pestivirus were detected in four animals (0.67%) from four different slaughterhouses (range at slaughterhouse level, 0.0–1.8%).

**Fig. 2:**
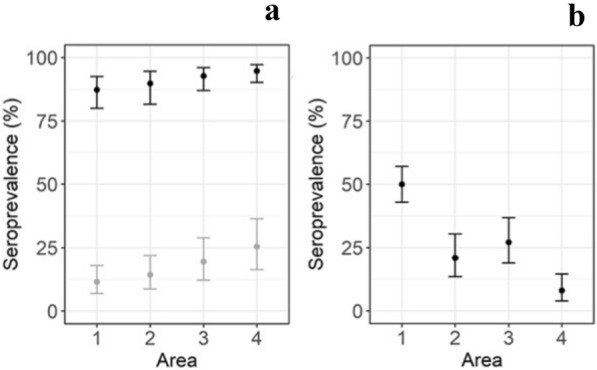
Predicted probability to find a seropositive sample against alphaherpesvirus (**a**) and gammaherpesvirus (MCFV-group) (**b**) in 596 semi-domesticated Eurasian tundra reindeer (*Rangifer tarandus tarandus*) sampled in 2015 in Finland, dependent on Region form south to north (Region 1–Region 4). Seroprevalence against alphaherpesvirus was significantly higher in adult reindeer (black) than calves (grey) but no age effect was observed for gammaherpesvirus. The presented values are pointwise back transformed using exp(^estimate^)/(1 − exp(^estimate^) * 100

Logistic regression models were run for testing positive for antibodies against alphaherpesvirus and MCFV. For having antibodies against alphaherpesvirus, the region and the age group were significant variables. There was a significantly higher seroprevalence of antibodies against alphaherpesvirus in the northern-most region (Region 4), having the highest animal density, as compared to the southern-most region (Region 1) (Figs. 1 and 2, Table 4). The probability to have a sample that was positive for antibodies against Alphaherpesvirus was 7.5 times higher in adult reindeer compared to calves. For MCFV the probability to have a positive sample was six times higher in the southern-most region (Region 1) as compared to the northern-most region (Region 4) (Figs. 1 and 2, Table 5). There was no significant difference in seroprevalence of antibodies against MCFV between calves (27.4%) and adults (31.5%).

**Table 4:** Logistic regression model estimates explaining the seroprevalence against alphaherpesvirus in semi-domesticated Eurasian tundra reindeer (*Rangifer tarandus tarandus*) sampled in Finland in 2015

Variable	Estimate	SE	Z	P
Adult Region 1 (Intercept)	1.93	0.29	6.70	< 0.001
Region 2	0.25	0.35	0.71	0.480
Region 3	0.62	0.33	1.89	0.059
Region 4	0.96	0.34	2.82	0.004
Calf	− 3.96	0.27	− 14.48	< 0.001

The explanatory variables included were Region (Region 1 to Region 4, from south to north) and age group (calf or adult)

**Table 5:** Logistic regression model estimates explaining the seroprevalence against gammaherpesvirus in semi-domesticated Eurasian tundra reindeer (*Rangifer tarandus tarandus*) sampled in Finland in 2015

Variable	Estimate	SE	Z	P
Region 1 (Intercept)	2.48e−15	0.15	1.70e−14	1.000
Region 2	− 1.33	0.26	− 5.09	< 0.001
Region 3	− 0.99	0.23	− 4.26	< 0.001
Region 4	− 2.43	0.36	− 6.75	< 0.001

The explanatory variable included was Region (Region 1 to Region 4, from south to north)

## Discussion

This study yielded new and needed baseline information on exposure to a selection of viruses among semi-domesticated reindeer in Finland. The results add to the knowledge of the presence, prevalence and epidemiology of these viruses which can be useful both for management and the future monitoring of reindeer health.

Strengths of this study include the large sample size allowing good estimates of seroprevalence, and the study design ensuring good geographical representation throughout the reindeer herding region of Finland. The sampling at slaughter ensured good quality of the samples. On the other hand, sampling slaughter animals does not necessarily represent a random sample from the total reindeer population in Finland even though the different reindeer regions are represented. Animals are selected for slaughter for different reasons (e.g., the sex structure of the herd, age and tooth wear). Thus, the slaughtered reindeer may not necessarily be representative for their donor herds and the results presented may be regarded as sample prevalence rather than population prevalence. Furthermore, it should be noted that the sampling for this study took place in 2015, and the epidemiological situation may have changed since then. The analyses are conducted by employing serological tests designed for similar viruses in livestock, introducing uncertainties whether the sensitivity and specificity of the tests are suitable for this purpose. Unfortunately, this is almost always the case when analyzing blood samples from free-ranging and wild animals. However, the tests used do have a multi-species design (e.g., competition) and have also to some extent been validated by other tests and have been used previously to test samples from reindeer. Nevertheless, the seroprevalence obtained in this study should be interpreted with care.

The results of this study indicated that alphaherpesvirus was enzootic in the Finnish reindeer population. This is in line with results from a similar screening of 618 semi-domesticated reindeer from eight herds in Norway [14]. Comparing these two investigations revealed a similar seroprevalence for alphaherpesvirus in Finland (46.5%, range 28.6–64.3%) and Norway (42%, range 21–62%). In both studies the seroprevalence was significantly higher in adult reindeer compared to calves, indicating that the risk of exposure to alphaherpesvirus increases with age, which is expected for a virus that establish life-long infections and latency [12].

The higher seroprevalence in the northernmost region (Region 4; 55.6%) compared to the southernmost (Region 1; 36.2%) may be a result of the higher reindeer density in the northern region (Table 3), being 2.41 reindeer/km^2^ for the northern herds (Region 4) compared to 1.21 reindeer/km^2^ for the southernmost herd (Region 1). However, the animal density as used here (animals/km^2^) do not necessarily account for available area versus used habitat. Furthermore, semi-domesticated reindeer are gregarious and roam in groups with temporal variation in group size associated with herd structure (sex and age), weather conditions, animal behavior, availability of pasture resources, disturbance (e.g., people, predators) and different herding regimes (e.g., feeding). Thus, animal count per km^2^ may not represent a reliable measure of the possibilities of a virus to spread within a herd, such as nose-to-nose contact, aerosol transmission and transmission via the environment. Thus, the lack of information on available pasture, pasture quality and how the animals have used the different pasture regions over time makes animal density as used here not feasible to be included in the statistical models.

Alphaherpesvirus, presumably CvHV2, seems to be common in most reindeer and caribou populations that have been investigated [6, 12, 17, 36]. Furthermore, it has been shown that CvHV2 infections can be reactivated by immunosuppression and stress [10, 37, 38] causing outbreaks of IKC and possibly other and more subtle health disorders [6, 8, 12]. Thus, given the broad geographical distribution in many subspecies and populations of *Rangifer*, CvHV2 infections may have larger health impacts on these populations than previously estimated.

The results also indicated that MCFV is enzootic in some regions in Finland, which again is similar to findings in Norway [14]. However, the seroprevalence in Finland (29.0%, range 3.5–62.2%) was almost three times as high as that in Norway (11%, range 2–15%) [14]. In contrast to the results for alphaherpesvirus, the prevalence of antibodies against MCFV was higher in the southernmost region (Region 1, 50.0%) compared to the northernmost region (Region 4, 8.1%). This observation is geographically similar to the recent findings in Norway, with a higher seroprevalence in the four southernmost sampling regions (Region 5–8; mean 14.7%) compared to the four northernmost sampling regions (Region 1–4; mean 6.3%) [14]. Results from screening 3339 semi-domesticated reindeer in Finnmark County, Norway, showed that the risk of exposure increased with increasing population density within the region [13]. This is in contrast to and opposite of the findings for the Finnish reindeer (this study), indicating that animal density alone may not be the only or major risk factor for exposure to MCFV. In the same screening (Finnmark, Norway), the seroprevalence was significantly higher for adults than for calves [13], whereas no significant age difference was found in the present study (Finland).

The MCFV cELISA used in this study is based on a viral epitope present in all viruses in the MCFV group, therefore it does not allow for identification of the specific MCFV infecting the animals. Based on the relatively high prevalence of seropositive animals, up to 62.2% in Region 1, with no report of clinical cases, it is possible that a reindeer specific MCFV that has not yet been identified is present in the population, or the reindeer may be sub-clinically infected with a known MCFV, most likely OvHV-2 and/or CpHV-2, which have been shown to infect cervids in Norway [11]. Alternatively, cross reactivity in the MCFV cELISA with Rangiferine gammaherpesvirus 1, a close relative of MCFVs recently reported in reindeer [39] may explain the seroprevalence observed in Finland. Follow up studies to test these hypotheses are needed to better inform about the impact of gammaherpesvirus infections in reindeer populations.

The prevalence of antibodies against Pestivirus (0.67%) was similar to recent findings in three Finnish herds (2016–2017; n = 122) showing a seroprevalence of 2.5% [15]. Both results are surprisingly low compared to recent findings in semi-domesticated reindeer in Norway showing a seroprevalence of 12–19% [14, 32] and in Sweden where a serological screening of 1158 semi-domesticated reindeer in 2001–2002 revealed a seroprevalence of 32% [7]. A more recent screening in Norway and Sweden confirmed higher seroprevalence estimates for the two countries, 41% in Norway and 49% in Sweden, based on samples from three different and geographically separated herds in both countries [15]. Although several serological screenings have documented exposure of wild and semi-domesticated reindeer and caribou to Pestivirus, no Pestivirus has been isolated from these populations. An experimental inoculation of reindeer with BVDV caused loose stool with blood and mucus, serous to mucopurulent nasal discharge and transient coronitis and laminitis [40]. Furthermore, a Pestivirus, designated V60, was isolated from a reindeer that suffered from diarrhoea and anorexia in a German zoo in 1996 [41, 42]. Virus characterization studies have shown that this virus was most closely related to Pestivirus D (BDV-2) [42, 43]. Although no clinical disease has been associated with Pestivirus infections in semi-domesticated reindeer under normal herding conditions or in wild reindeer, the findings from the case in a zoo and the experimental inoculation suggest that reindeer are susceptible to Pestivirus infections. The high seroprevalence in certain herds and regions further suggests that Pestivirus infections may impact the health status of reindeer, which should be further investigated.

No antibodies were detected against BTV or SBV, and no exposure of semi-domesticated or wild reindeer to these viruses has so far been reported in the available literature. However, when screening Eurasian tundra reindeer in zoos in Germany (2013; n = 118) a seroprevalence of 3.4% and 59% was found against BTV and SBV, respectively, indicating that reindeer are susceptible to these infections [33]. This should be kept in mind should BTV again increase its distribution in cervid populations in Europe.

We have revealed that Finnish reindeer are exposed to several infectious agents that are pathogenic to reindeer. Clinical cases and sometimes disease outbreaks are shedding light on the importance of such infections, but it is often challenging to evaluate their overall impact, especially on population level. Furthermore, we know that the presence and exposure to a pathogen may not alone elicit a disease outbreak, that disease outbreaks may be associated with stress (e.g., transport, feeding) and that pathogens may operate together, causing several and different disease outbreaks in the same herd, such as CE (ORFV), IKC (CvHV-2) and necrobacillosis (*F. necrophorum*) [44].

A changing climate, such as rain-on-snow events that may cause ice-locked pastures, and encroachment of pastureland have initiated mitigating actions in reindeer herding [1]. This may further affect the level of exposure to viruses and other pathogens in the future. For example, the practice of feeding reindeer, especially if done in corrals and for extended periods of time, may facilitate transmission of infectious agents between animals due to increased nose-to-nose contact, aerosol transmission and poor hygienic conditions at gathering spots [44, 45]. Intensive feeding may also represent a closer link between livestock and semi-domesticated reindeer due to e.g., sharing of feed, feed production lines, corrals, animal transport and equipment. Thus, the presence, prevalence and exposure to reindeer pathogens is expected to change over time, necessitating continuous surveillance of health parameters of this species.

## Conclusions

The results of this study add to the knowledge on reindeer health and on the presence, prevalence and epidemiology of the viruses investigated. Comparable to Norway and Sweden, alphaherpesvirus and gammaherpesvirus (MCFV) seems to be enzootic in Finnish reindeer, whereas the seroprevalence against Pestivirus was surprisingly low in Finland as compared to reindeer in Norway and Sweden. These results constitute a reference baseline information that is useful to address future changes in the reindeer herding industry in Finland.

## Data Availability

The datasets used and/or analyzed during the current study are available from the corresponding author upon request.
